# Metagenomic Survey of the Highly Polyphagous *Anastrepha ludens* Developing in Ancestral and Exotic Hosts Reveals the Lack of a Stable Microbiota in Larvae and the Strong Influence of Metamorphosis on Adult Gut Microbiota

**DOI:** 10.3389/fmicb.2021.685937

**Published:** 2021-08-02

**Authors:** Martín Aluja, Jesús Alejandro Zamora-Briseño, Vicente Pérez-Brocal, Alma Altúzar-Molina, Larissa Guillén, Damaris Desgarennes, Mirna Vázquez-Rosas-Landa, Enrique Ibarra-Laclette, Alexandro G. Alonso-Sánchez, Andrés Moya

**Affiliations:** ^1^Red de Manejo Biorracional de Plagas y Vectores, Instituto de Ecología, AC–INECOL, Clúster Científico y Tecnológico BioMimic^®^, Xalapa, Mexico; ^2^Fundación para el Fomento de la Investigación Sanitaria y Biomédica de la Comunitat Valenciana (FISABIO), Valencia, Spain; ^3^Red de Biodiversidad y Sistemática, Instituto de Ecología, AC–INECOL, Clúster Científico y Tecnológico BioMimic^®^, Xalapa, Mexico; ^4^Red de Estudios Moleculares Avanzados, Instituto de Ecología, AC–INECOL, Clúster Científico y Tecnológico BioMimic^®^, Xalapa, Mexico; ^5^Instituto de Biología Integrativa de Sistemas (I^2^Sysbio), Universidad de Valencia-CSIC, Valencia, Spain

**Keywords:** microbiota, plant-insect interactions, *Anastrepha ludens*, Tephritidae, gut

## Abstract

We studied the microbiota of a highly polyphagous insect, *Anastrepha ludens* (Diptera: Tephritidae), developing in six of its hosts, including two ancestral (*Casimiroa edulis* and *C. greggii*), three exotic (*Mangifera indica* cv. Ataulfo, *Prunus persica* cv. Criollo, and *Citrus* x *aurantium*) and one occasional host (*Capsicum pubescens* cv. Manzano), that is only used when extreme drought conditions limit fruiting by the common hosts. One of the exotic hosts (“criollo” peach) is rife with polyphenols and the occasional host with capsaicinoids exerting high fitness costs on the larvae. We pursued the following questions: (1) How is the microbial composition of the larval food related to the composition of the larval and adult microbiota, and what does this tell us about transience and stability of this species’ gut microbiota? (2) How does metamorphosis affect the adult microbiota? We surveyed the microbiota of the pulp of each host fruit, as well as the gut microbiota of larvae and adult flies and found that the gut of *A. ludens* larvae lacks a stable microbiota, since it was invariably associated with the composition of the pulp microbiota of the host plant species studied and was also different from the microbiota of adult flies indicating that metamorphosis filters out much of the microbiota present in larvae. The microbiota of adult males and females was similar between them, independent of host plant and was dominated by bacteria within the Enterobacteriaceae. We found that in the case of the “toxic” occasional host *C. pubescens* the microbiota is enriched in potentially deleterious genera that were much less abundant in the other hosts. In contrast, the pulp of the ancestral host *C. edulis* is enriched in several bacterial groups that can be beneficial for larval development. We also report for the first time the presence of bacteria within the Arcobacteraceae family in the gut microbiota of *A. ludens* stemming from *C. edulis*. Based on our findings, we conclude that changes in the food-associated microbiota dictate major changes in the larval microbiota, suggesting that most larval gut microbiota is originated from the food.

## Introduction

The critical role the microbiome plays in supporting or outright regulating key metabolic pathways in organisms including humans or in dealing with emerging environmental challenges is being unraveled at an unprecedented speed (Gevers et al., 2012; Gilbert et al., 2014; Bauer et al., 2020; Ayyasamy et al., 2021; Schmidt and Engel, 2021). Although the role that microorganisms play in the life cycle of many organisms became already clear over 100 years ago (Petri, 1909) the interdependency and sophistication of the interactions, as well as their evolution and impact on overall host health and fitness, is being comprehensively investigated by the scientific community worldwide (Sommer and Bäckhed, 2013; Rowland et al., 2018; Jing et al., 2020; Ke et al., 2020). In the case of insects, it has been demonstrated that the gut microbiota plays multiple roles in nutrient uptake (e.g., the synthesis and absorption of nutrients), detoxification processes (Kikuchi, 2009; Ben-Yosef et al., 2015; Ceja-Navarro et al., 2015; Cheng et al., 2017), sexual fitness, health maintenance or longevity, and overall host physiology (Brummel et al., 2004; Dillon and Dillon, 2004; Engel and Moran, 2013; Pernice et al., 2014; Damodaram et al., 2016; Sudakaran et al., 2017; Jose et al., 2019; Raza et al., 2020). As insects are the most diverse group of animals on Earth, with over one million species (Grimaldi and Engel, 2005), they are considered as an ideal group to study the different degrees of association between the gut microbiota and their hosts (Hammer et al., 2019; Muñoz-Benavent et al., 2020), even if the complexity of the insect gut microbiota is far less complex than that of mammals (Sender et al., 2016).

As is the case in many other animals, the gut microbiota of insects can be classified as resident or transient. Some of these microorganisms were discovered during the beginning of the last century (Glasgow, 1914; Stammer, 1929) and may switch from an intra to an extracellular existence during host development (Estes et al., 2009). The resident microbiota is the microbial community that establishes long-lasting interactions with the host (Derrien and van Hylckama Vlieg, 2015; Hammer et al., 2017, 2019; Ma and Leulier, 2018) and can be of two types: (1) Obligatory endosymbionts embedded inside cells, that have taken over specific metabolic functions and are heritable and transmitted vertically (Akman et al., 2002; Baumann et al., 2002; Baumann, 2005) or that have become unwelcome parasites, such as is sometimes the case with *Wolbachia*, that can harm the host by, for example, controlling sex ratios (Werren et al., 2008; Fukui et al., 2015; Jiggins, 2016). (2) A large and diverse suite of microorganisms, particularly bacteria, yeasts, and viruses, that are found in the gut of both adults and larvae, and that are transmitted vertically or horizontally [i.e., are taken up from the environment and thus greatly vary in species composition according to environment, diet, age, physiological state, and health status (Engel and Moran, 2013; Johnston and Rolff, 2015; Pérez-Cobas et al., 2015; Muñoz-Benavent et al., 2020)]. In contrast to resident microbiota, the transient microbiota is exclusively acquired from the environment and usually passes through the gut and is expelled via the feces. It is not capable of establishing itself permanently, since its rate of loss exceeds its rate of permanence (Erkosar and Leulier, 2014; Derrien and van Hylckama Vlieg, 2015; Ma and Leulier, 2018; Hammer et al., 2019).

In general, those species with a stable gut microbiota closely rely on the functions provided by it. In contrast, the alteration of the gut microbiota has little influence on survival and development in insects with an unstable gut microbiota (Hammer et al., 2019). A direct relationship has been suggested between the metabolic self-sufficiency of the host and the absence of a stable gut microbiota (Hammer and Bowers, 2015; Hammer et al., 2017), supporting the idea that a stable gut microbiota is not equally important for all insect species (Hammer et al., 2019; Phalnikar et al., 2019). For example, the lack of a stable microbiota in certain caterpillars is related to the fact that caterpillars possess host-encoded mechanisms for degrading or tolerating plant allelochemicals (Després et al., 2007). In addition, it has been proposed that the lack of a stable gut microbiota could constitute an adaptive strategy, through which certain species can avoid establishing permanent interactions with microorganisms (Hammer et al., 2019).

Alternatively, other studies support the critical role gut microbiota plays on the physiology, adaptation, or ecological interactions of insects (Janson et al., 2008; Kageyama et al., 2012; Killiny et al., 2017; Gupta and Nair, 2020; Lemoine et al., 2020). For example, there is evidence that during plant-insect interactions, the gut microbiota can play a key role degrading plant structural compounds and supplementing missing nutrients, but also detoxifying deleterious compounds of chemically defended plants that they attack, by providing counter-defenses to plant toxins (Dowd, 1989; Douglas, 2009; Hansen and Moran, 2014; Hammer and Bowers, 2015). Based on this, Hammer and Bowers outlined the gut microbial facilitation hypothesis, which proposes that “variation among herbivores in their ability to consume chemically defended plants can be due, in part, to variation in their associated microbial communities” (Hammer and Bowers, 2015). One direct consequence of this hypothesis is that the gut microbiota may contribute to rapidly expanding the range of plants that insects can consume and assimilate.

Excluding termites, most recent work on insect microbiota has concentrated on cockroaches (Ayayee et al., 2018; Kakumanu et al., 2018; Guzman and Vilcinskas, 2020), beetles (Tsuchida et al., 2010; Zepeda-Paulo et al., 2010; Guyomar et al., 2018; McLean et al., 2019), and Drosophila (Drosophilidae) (Wong et al., 2013; Douglas, 2018; Ma and Leulier, 2018; Pais et al., 2018; Selkrig et al., 2018; Ludington and Ja, 2020). In the case of true fruit flies (Tephritidae), there are some classical studies on the interaction of them with microorganisms, but most of this research was focused on a few species within the genera *Anastrepha*, *Bactrocera*, *Ceratitis*, and *Rhagoletis*. For example, Stammer (1929) compared 37 species within the Tephritidae (formerly Trypetidae) in 24 genera, finding in almost all cases close associations between the flies and bacteria. Allen et al. (1934) described consistent associations between *Phytomonas* (*Pseudomonas*) *melophthora* and the apple maggot, *Rhagoletis pomonella* (Walsh), discussing the possibility that the female was contaminating the fruit with the bacteria while ovipositing, a broad topic retaken by Mazzini and Vita (1981), Stoffolano and Yin (1987), Raghu et al. (2002) and Behar et al. (2008). By far the best studied genus is *Bactrocera* in which, bacteria (Fitt and O’Brien, 1985; Capuzzo et al., 2005; Estes et al., 2009, 2014; Ben-Yosef et al., 2015; Yong et al., 2017; Heys et al., 2018; Liu et al., 2018; Akami et al., 2019), yeasts (Deutscher et al., 2017; Piper et al., 2017), fungi (Malacrinò et al., 2017; Yao et al., 2019), and viruses (Zhang et al., 2020), have been studied.

The case of *B. oleae* and its symbiotic interaction with bacteria represents an instructive example on the importance of the gut microbiota in allowing the host use of chemically defended plants (i.e., *Olea europaea* L.). In this fruit fly (formerly *Dacus oleae*), Petri (1904, 1909) was the first to describe a guild of larval gut bacteria, highlighting *Bacterium* (*Pseudomonas*) *savastanoi* and *Ascobacterium luteum*. He emphasized the relevance of *B*. *savastanoi* in possibly providing critical nutrients to the fly, also hinting at the bacterial breakdown of deleterious substances for the larvae, as well as dwelling on its importance in the immune system, by keeping *A*. *luteum* at bay and defending against infections produced by fungi and bacteria. Petri (1909) observed that if *B*. *savastanoi* was not present in the tract of *B*. *oleae* feeding in fruit of *O*. *europaea*, development was halted and individuals died, concluding that there was an obligatory symbiosis between the bacteria and the host, and that the bacteria were vertically transmitted from the female to the larvae via the “contaminated” eggs. Then, Hagen (1966) performed a series of experiments with streptomycin to demonstrate that *B. oleae* larvae need the bacteria to detoxify deleterious chemicals contained in unripe olives. He inferred that *B. savastanoi* “may be involved in the synthesis of methionine and threonine,” a topic also studied by Miyazaki et al. (1968) working with *Pseudomonas melophthora* and *R. pomonella*. More recently, Yuval et al. highlighted the central role the fruit plays with respect to fruit fly-bacteria interactions in which the latter aid in nitrogen fixation (Behar et al., 2008) and unraveled the specific role of *Candidatus* Erwinia dacicola [formerly referred t*o as B. savastanoi* (Capuzzo et al., 2005)] in dealing with the toxic allelochemical oleuropein (a phenolic glycoside) present in unripe olives on which larvae feed (Ben-Yosef et al., 2015; Sinno et al., 2020), confirming the visionary suggestions previously made by Petri (1910) and Hagen (1966).

Based on all the above, but pointedly also motivated by the outstanding questions posited by Frago et al. (2012), Hansen and Moran (2014), Hammer and Bowers (2015), Hammer et al. (2017), Sudakaran et al. (2017), and Majumder et al. (2019), here we aim at providing a more in-depth ecological context to insect gut microbiota studies by comparing the microbiota in the gut of larvae reared in six hosts in nature of the highly pestiferous and polyphagous tephritid fly, *Anastrepha ludens* (Loew), also known as the Mexican Fruit Fly. We selected the two purportedly ancestral native hosts of *A. ludens* [*Casimiroa greggii* (S. Watson) F. Chiang and *Casimiroa edulis* La Llave (both Rutaceae)], an additional native one [*Capsicum pubescens* cv. Manzano (Solanaceae)], as well as three exotic ones [*Citrus x aurantium* (Rutaceae), *Mangifera indica* cv. Ataulfo (Anacardiaceae), and *Prunus persica* cv. Criollo (Rosaceae)], ranging from commonly used fruit (e.g., *C*. x *aurantium*) to rarely used hosts (e.g., *C*. *pubescens*) so as to render our comparison more robust and ecologically meaningful (Aluja and Mangan, 2008). Birke and Aluja (2018) working with *A*. *ludens* and almost the same hosts we studied here, report that pupal weights (mg) of larvae developing in these fruits were: *C. edulis* 23.02 ± 0.19, *M. indica* 21.25 ± 0.16, *C. aurantium* 18.23 ± 0.15, *C. pubescens* 16.12 ± 0.35, and *P. persica* 11.55 ± 0.25 mg, respectively. That is, there is a significant fitness cost when *A*. *ludens* infests *C*. *pubescens* cv. Manzano and *P*. *persica* cv. Criollo, suggesting the presence of potentially toxic capsaicinoids (Manzano pepper) and polyphenols (Criollo peach). Given that we were principally interested in determining bacterial diversity associated with host plants and the possible role of the microbiota in host exploitation, we concentrated our effort in determining bacteria in fruit pulp (tissue in which larvae had naturally developed), gut of larvae and freshly emerged adults stemming from the latter larvae. We pursued the following questions: (1) How is the microbial composition of the larval and adult microbiota related to the composition of food microbiota? and (2) what does this tell us about transience and stability of this species’ gut microbiota from larvae to adult metamorphosis? Based on the extreme polyphagy of *A*. *ludens*, we hypothesized that independent of host, we would find a core microbiota aiding the fly in the digestion of multiple substrates encountered by the larvae in the pulp of the many hosts into which a female lays eggs over the various fruiting seasons along the year.

## Materials and Methods

### Hosts and Collection Sites

**FIGURE 1:**
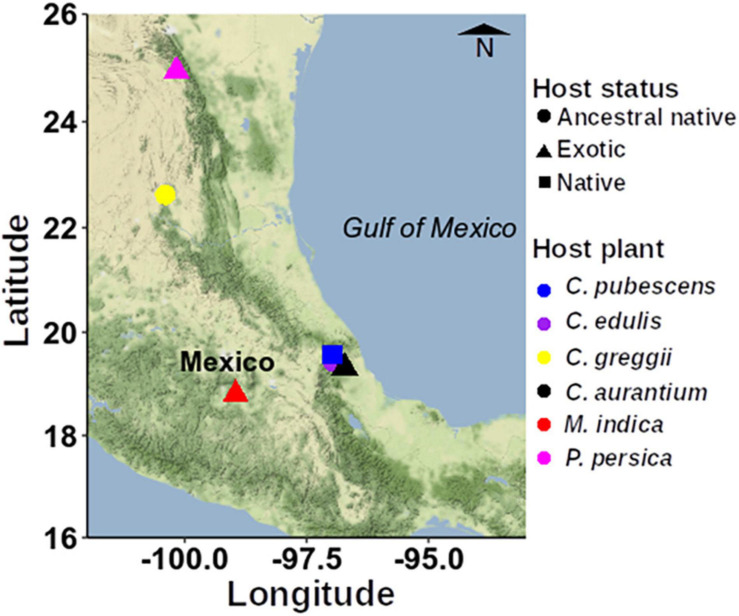
Map showing the sampling sites within Mexico used to collect the infested fruits. To construct this figure, we used the ggmap spatial visualization package (Kahle and Wickham, 2013).

The six hosts selected are representative of the wide array of fruit exploited by *A*. *ludens* over its geographic range [S Texas to Costa Rica (Norrbom et al., 2000)]. We collected naturally infested fruit mainly in the State of Veracruz, Mexico but also in the States of San Luis Potosí, Nuevo León and Morelos (Figure 1 and Supplementary Table 1).

### Sample Processing

In the field, individual overripe fruit of the six *Anastrepha* hosts studied (details in Figure 1), with signs of infestation by fruit fly larvae (e.g., breathing/exit orifices) were collected while still attached to branches and immediately dissected on site to obtain pulp and larva samples. We cut open the fruit with a sharp, sterile knife, and in the vicinity where third instar larvae were detected, a sample of ca. 0.5 g of pulp was obtained with a sterile spatula. A caveat related to this sampling approach, is that we cannot rule out the possibility that the pulp in the vicinity of where larvae were feeding was not contaminated by bacteria present in the eggs or the feces of larvae and recognize that ideally, we should have compared this pulp from infested fruit with pulp from uninfested fruit stemming from the same tree to conclusively determine if indeed the presence of larvae contaminated the undamaged pulp. But as females are highly selective when choosing fruit to lay their eggs, we thought that sampling pulp from uninfested fruit would have biased our results as there was the possibility that undamaged fruit surrounding the infested fruit were somehow unsuitable based on the decision made by the female (Birke and Aluja, 2018).

Samples were immediately transferred to a 2 mL vial and frozen in liquid nitrogen. In addition, we fetched five larvae crawling in the pulp with the help of sterile tweezers. As was the case with pulp samples, larvae were individually placed in 2 mL vials, and immediately frozen in liquid nitrogen. Before DNA extraction, larvae were individually surface sterilized using a sterile washing solution (SDS 1%, *Tris* 10 mM, and NaCl 10 mM) for 1 min, 1% commercial bleach for one min, 70% ethanol for one min and finally, two 1-min washes with sterile distilled water.

Additional fruit in the same condition and collected directly from the tree were transported back to the laboratory (some field sites where over 700 km away) and handled in 10 L plastic containers into which a 1-cm thick layer of sterile vermiculite was placed in the bottom part of the container. This allowed any larvae inside fruit to exit it and crawl into the vermiculite to pupate. Once back in the laboratory, vermiculite or any pupae that were already formed were sprayed with sterile distilled water until they emerged as adults. Newly emerged adults were dissected under sterile conditions to isolate the complete digestive system, from the cardia to the anus. Ten adult gut samples from specimens emerged from each of the six fruits studied were stored in RNA later and preserved at −80°C until their analysis.

### DNA Extraction, 16S Gene Amplification and Sequencing

Each pulp DNA extract represented a combined pool of five 20 mg pulp samples, each larval DNA extract stemmed from a single larva, and each adult DNA extract represented a combined pool of two adult guts. We used five replicates per sample type (pulp, larva, or gut). Total DNA was extracted using QIAamp DNA Mini Kit (Qiagen©, Hilden, Germany). Amplicons of the variable V3 region of the 16S gene were amplified from 100 ng of total DNA samples using the primers described by Klindworth et al. (2013). PCRs (50 μL) containing Qiagen© buffer 1X, dNTPs 0.2 mM, 16s F&R 0.2 μM, Taq polymerase 2.5 U were performed using the following amplification program: 94°C/2 min of initial denaturation followed by 25 cycles of 94°C/15 s denaturation, 55°C/30 s annealing, and 72°C/1 min amplification, and a final amplification at 72°C/5 min. Illumina libraries were constructed by adding Nextera XT adapters (Illumina Inc.©). Initial amplification and adapter-ligation PCRs were performed using Kapa polymerase® (Kappa Biosystems©) and purified immediately after finishing each PCR using 0.8X Agencourt Ampure XP cleaning beads® (Beckman Coulter©). Library concentration was quantified using a DNA HS kit (Invitrogen©) in a Qubit 2.0® fluorometer (Invitrogen©). Library size was evaluated using a Bioanalyzer DNA HS® chip (Agilent©). Libraries were diluted and pooled to an equimolar concentration to be denatured and loaded into a MiSeq® sequencer using a MiSeq 600-cycle Reagent kit V3® (Illumina Inc.©). Sequencing procedures were performed at INECOL’s sequencing unit in the BioMimic® Scientific and Technological Cluster.

### Bioinformatic Analyses

Raw sequences were processed using the DADA2 package to resolve the amplicon sequence variants (ASVs) (Callahan et al., 2016). We included the following filter criteria: (i) cut of the sequences at 280 and 230 bp, of the sense and antisense reads, respectively; (ii) an error threshold of one biased assigned base in sense reads and two in antisense reads, respectively; and (iii) deletion of sequences with ambiguous bases. From the filtered sequences, error modeling was performed (Callahan et al., 2016). The paired sequences were merged and filtered to remove chimeric sequences using the “removeBimeraDenovo” algorithm with the “consensus” method (Callahan et al., 2016). Sequences were used to construct merged sequences. Then, the taxonomic assignment was performed with DECIPHER 2.14.0 (Wright, 2016) using the SILVA database version 138 (Quast et al., 2013). Sequences that were unidentified, those with a relative abundance of less than 1%, and those identified as “Mitochondria” and “Chloroplast” were removed at the phylum level (Callahan et al., 2016). The feature table was standardized to the number of sequences of the smallest library. The statistical analyses were performed with the phyloseq (McMurdie and Holmes, 2013), ggplot2 (Ginestet, 2011), ggalt^1^, and vegan (Oksanen et al., 2008) packages.

To test whether the microbiota differed between adult males and females, we assessed the Beta diversity of the bacterial communities between males and females using a PERMANOVA analysis (pairwise test with 1,000 permutations), based on Bray-Curtis similarities. The Shannon diversity indices were calculated, and statistical significance was estimated using a paired *t*-test with an α = 0.05. Networks were calculated running the make_network function of the phyloseq package initially using the object phyloseq. Results were graphed with the function plot_network. For the make_network function we used the following parameters: max.dist = 0.9, dist.fun = “jaccard.” The parameter max.dist defined the maximum ecological distance allowed between two samples to still be able to consider them connected by an “edge” in the graphic model. For the plot_netword function, we specified the following parameters: color = “origin,” shape = “host,” line_weight = 0.2, label = NULL.

Non-metric multidimensional scaling (NMDS) with the unweighted unifrac distance was applied to the data to assess grouping by host plant among the microbiota of the pulp, larvae, and adults. In each case, we drew grouping lines, which were obtained with the geom_encircle function of the ggalt package (see footnote text 1). To visualize the top-most representative genera, we plotted those genera that represent more than 10% of the relative abundance per host plant. The core microbiota, or the set of amplicon sequence variants detected in 50–100% (prevalence) of the samples with a relative abundance threshold value above 0.01%, was identified using the core function in the microbiome R package version 1.5.28 (Leo and Sudarshan, 2017). A linear discriminant analysis (LDA) effect size (LEfSe) method was applied to identify taxonomic biomarkers, which were performed with the microbial^2^ package. All analyses were performed in the R environment (“R Team^3^”), version 3.6.3.

All raw sequence data were deposited in the NCBI Short Read Archive under the Bio project PRJNA715941.

## Results

The number of raw sequences per sample, after filtering, merging, and removing chimeras is shown in Supplementary Table 2. Across all the samples, ASVs identified were distributed among 13 bacterial phyla, 96 families, and 190 genera. The rarefaction curves constructed to estimate the extent of the analysis showed that the curves of all libraries reached the plateau phase or almost did so, supporting the depth of the sequencing effort (Supplementary Figure 1).

**FIGURE 2:**
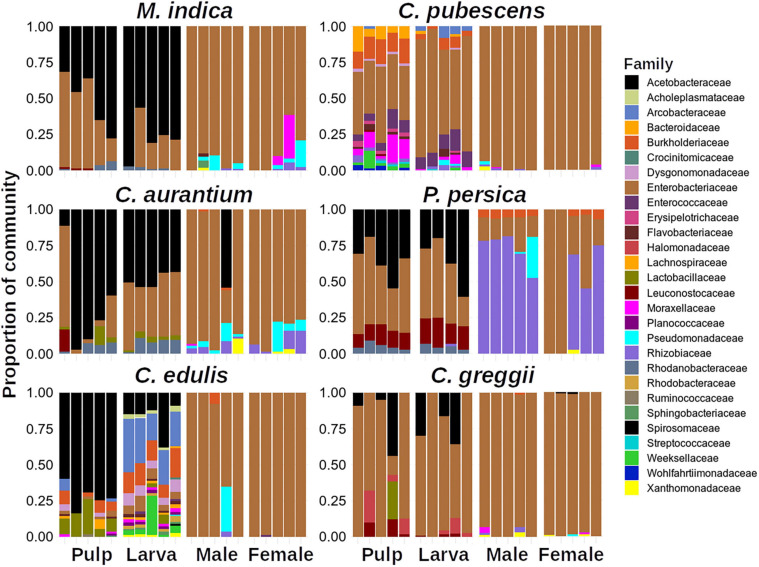
Relative abundances of Amplicon Sequence Variants (ASVs) among host plants at family level. Note that little differences are observed between males and females.

**FIGURE 3:**
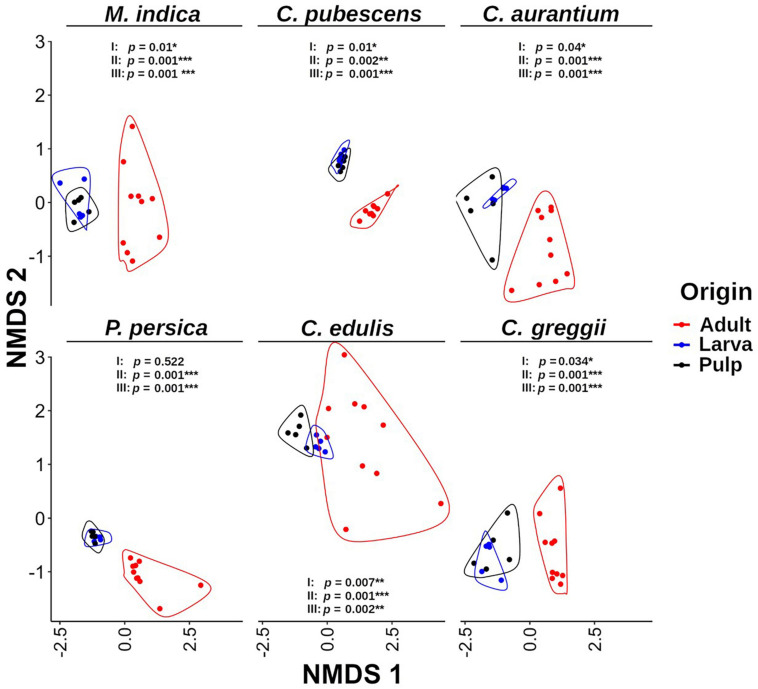
Non-metric multidimensional scaling (NMDS) analysis of the microbiota of pulp, larvae, and adults within host plant. It is evident that the gut microbiota of larvae is grouped with the pulp microbiota and both are separated from the microbiota of adult samples. The *p*-value corresponds to the results of the PERMANOVA test. I: larva vs. pulp; II: larva vs. adult; III: pulp vs. adult. Grouping lines were performed with the geom_encircle function of the ggalt package.

Figure 2 shows the relative abundances at the family level (details in Supplementary Table 3). In this figure, only taxa with >1% of relative abundance are shown. It is evident that the relative composition profile of the larvae was more like the one observed in the pulp but dissimilar to the adult (male or female) stage and that the structure was simpler in adults than in larvae and pulp (Figures 2, 3). Moreover, males and females exhibited very similar composition profiles with the Enterobacteriaceae being clearly dominant, with exception of adults emerged from *P. persica*, in which the Rhizobiaceae family was also dominant. In pulp and larvae, Acetobacteraceae and Enterobacteriaceae were dominant, except in *C. edulis*, where no clear dominance was observed.

As the relative abundance of taxa at the family level between males and females was quite similar, we decided to perform a statistical comparison between the microbiota of both sexes. In the comparison of male versus female gut microbiota, the PERMANOVA analysis detected no statistically significant differences between males and females [df = 1, *F* = 0.70247, *R*^2^ = 0.01197, Pr(>*F*) = 0.893; Table 1]. The data from females and males were therefore grouped under the category of “adults” for the comparisons of the microbiota of the pulp, larvae, and adults by host plant.

**TABLE 1:** PERMANOVA analyses of the bacterial communities associated with *Anastrepha ludens* considering all factors (i.e., host species, larvae, and fruit pulp).

Factor^a^	*F*	*R* ^2^	*p*
**Global**			
Host^5,113^	5.0669	0.18314	0.001*
^b^Origin^3,115^	3.5945	0.08573	0.001*
**Adults**			
Host^5,54^	5.1793	0.32413	0.001*
^C^Origin^1,58^	0.70247	0.01197	0.888
**Larvae^5,24^**			
Host	8.7892	0.64678	0.001*
**Pulp^5,24^**			
Host	6.1654	0.56226	0.001*

*^*a*^Subscript numbers indicate the degrees of freedom and residuals of each *F* test.*

*^*b*^Pulp, male, female, and larvae.*

^*c*^Males and Females.

*Significant factors at p ≤ 0.05.

**FIGURE 4:**
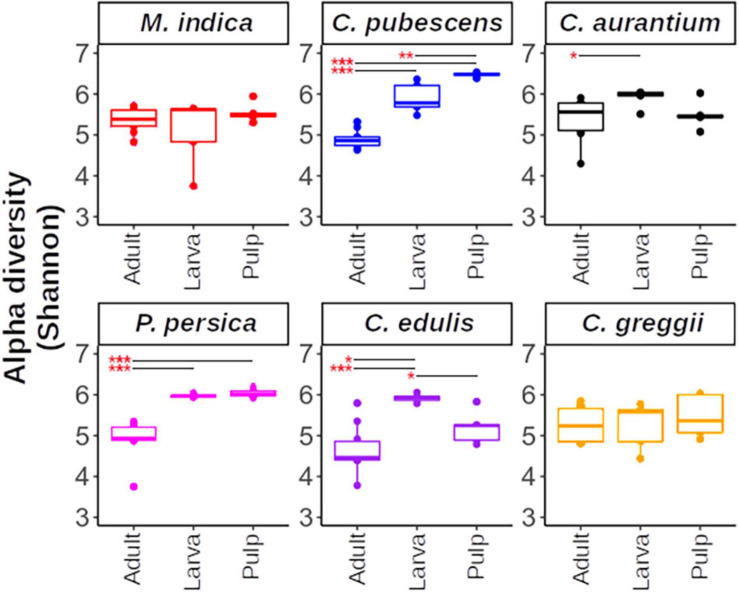
Alpha diversity within host plants estimated using the Shannon index. Upper and lower whiskers, 3rd, and 1st quartiles (top and bottom of the box), and median (horizontal line within the box) are displayed. Asterisks indicate significant differences between the pairs connected by the horizontal line. For paired *t*-test, *, **, *** represent *p* < 0.05, 0.01, and 0.001, respectively.

In general, within each host plant, adults, pulp, and larvae exhibited similar alpha diversity with *C. pubescens* being a notable exception (Figure 4). The pulp of this fruit had a more diverse microbiota than the larvae and adults. Interestingly, the gut microbiota of the larvae that developed in *C. edulis* had a more diverse microbiota than the pulp or adults. The comparisons of alpha diversity flushed out large differences in pulp, larval, and adult microbiota among host plant species (Supplementary Figure 2). Notably, the pulp microbiota of *C. pubescens* was the most diverse while the pulp of *C. edulis* was the least diverse. The microbiota of larvae that developed in *C. pubescens* was also the most diverse, while the microbiota of larvae that developed in *C. greggii* was the least diverse. In general, differences in adult microbiota among host species were less pronounced than in pulp and larvae.

**FIGURE 5:**
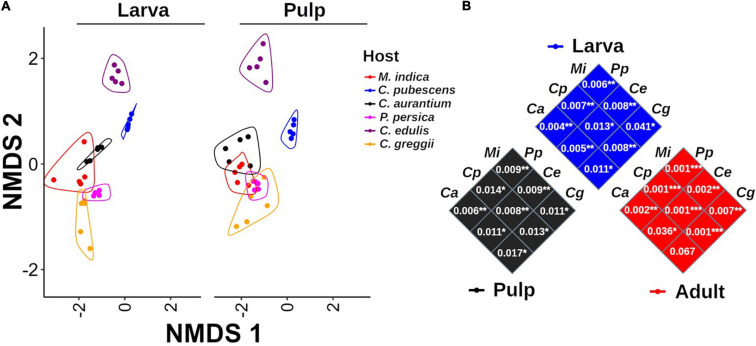
Non-metric multidimensional scaling analysis of the microbiota of pulp and larvae including all host species. **(A)** The microbiota of pulp and larvae exhibit a highly similar pattern among all host species. Grouping lines were drawn with the geom_encircle function of the ggalt package. **(B)** PERMANOVA comparisons among samples per origin (pulp, larva, and adult). *P*-values of the paired comparison resulting after running the PERMANOVA test are shown inside each square. *Mangifera indica* (Mi), *C. x aurantium* (Ca), *Casimiroa edulis* (Ce), *C. greggii* (Cg), *Capsicum pubescens* (Cp), and *Prunus persica* (Pp).

Based on the NMDS analysis of the Bray-Curtis distances of the microbiota of the pulp, larvae, and adults within each host plant species, the microbiota of the pulp and the larva were grouped together in each of the hosts and were separated from the adult microbiota (Figure 3). Except for *P*. *persica*, the PERMANOVA test indicated that the microbiota of the fruit pulp was different from the microbiota of the larvae in all host plants. However, it is worth mentioning that these differences were statistically less pronounced when compared to the differences detected among the microbiota of the pulp or the larva and the microbiota of the adult (Figure 3). When we compare the NMDS of larvae and pulp considering all host species, it also becomes clear that the microbiota of the larvae was related to the microbiota associated with the pulp the larvae fed on in all cases (Figure 5A). Importantly, in almost all cases, the microbiota of the pulp, larva and adults differed among host plants (Figure 5B).

**FIGURE 6:**
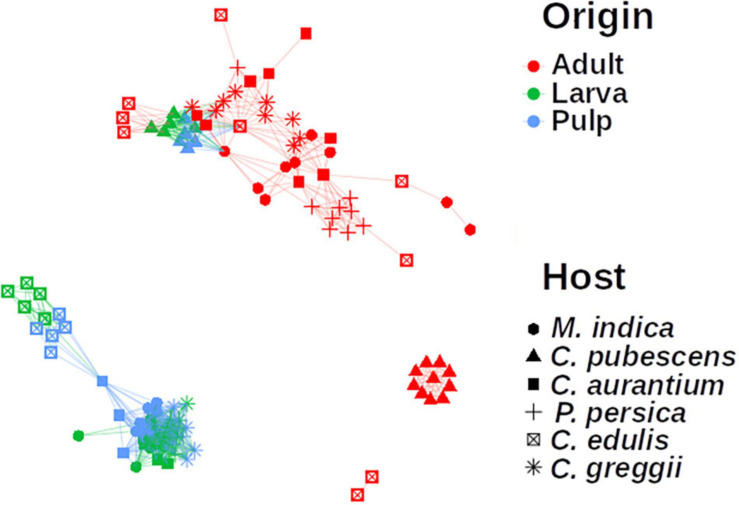
Network analysis of the microbiota of pulp, larvae, and adults, including all plant host species. The microbiota of pulp and larvae clustered together for all samples. Nodes represent the microbiota of each sample. Links between the nodes indicate significant correlations. The colors represent the origin the sample came from and the shape represents which host the microbiota is related to. The microbiota of the pulp and larvae of *C. edulis* and *C. pubescens* are grouped separately.

In the network analyses we observed that samples clustered according to the origin of the microbiota (adult, larvae, and pulp) and its host (Figure 6). The pulp microbiota clearly separated into three groups: (i) *C. edulis*, (ii) the hosts in which the fly develops well (the other native and all exotic hosts) and (iii) *C. pubescens*. The microbiota of the larvae was well grouped with the microbiota of the pulp in all cases and the microbiota of the adults was different from the microbiota of the pulp and larvae.

Since the microbiota of larvae developing in *C. edulis* and *C. pubescens* were clear outliers with respect to the microbiota of the larvae of the other host species analyzed, we compared the microbiota of larvae that developed in these two fruits with that of the other host plants to explore their differential taxa (Supplementary Figure 3). Based on this analysis, the family Acetobacteraceae was underrepresented in *C. pubescens* compared to the microbiota of the rest of the host species, while the genera *Providencia, Morganella*, and *Vagoccocus* were over-represented. In *C. edulis*, the Weeksellaceae, Acholeplasmataceae, Arcobacteraceae (newly reclassified group with interesting characteristics), Burkholderiaceae families, and the *Empedobacter*, *Lampropedia*, and *Dysgonomonas* genera were over-represented compared to the remaining host plants.

**FIGURE 7:**
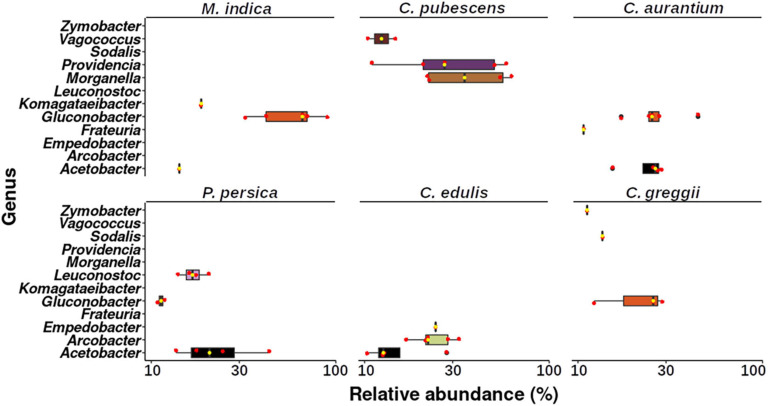
Boxplot showing the genera that represent more than 10% of the relative abundance in each larva sample per host plant. Upper and lower whiskers, 3rd and 1st quartiles (top and bottom of the box), and median (horizontal line within the box) are displayed. Median is also indicated as a yellow dot. Red dots represent the individual values of each sample.

**FIGURE 8:**
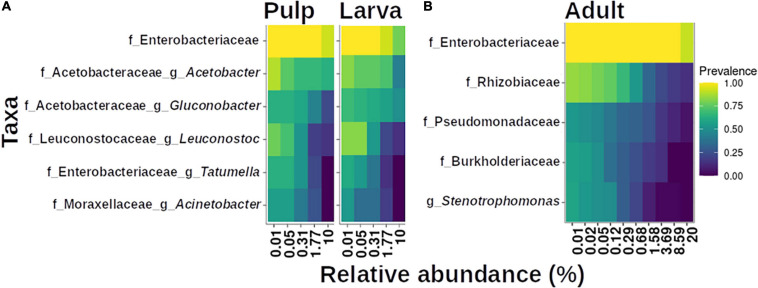
Core microbiota analysis based on relative abundance and sample prevalence of bacterial genera in pulp, larvae **(A)**, and adult **(B)** gut microbiota. For this analysis, the Amplicon Sequence Variants (ASVs) were agglomerated at the genus level. Only genera that were present in at least 50% of the samples within each category are displayed.

Finally, we found that the microbiota of the larva was strongly influenced by the host it developed in, which becomes evident when we observe the topmost abundant taxa among the larvae microbiota from the different host plants (Figure 7). The size and composition of the core microbiota of the larva varies according to the host species (Figure 8). Therefore, we integrated the data of all hosts into this analysis and found that the estimated core microbiota of the larvae was more diverse than the core microbiota of newly emerged adults, and it is equivalent to that of the pulp. Most of the bacteria identified as part of the adult core microbiota belonged to the Enterobacteriaceae, and to a lesser extent to the Rhizobiaceae, Pseudomonadaceae, and Burkholderiaceae families and the *Stenotrophomonas* genera. Notably, except for the genera within the Enterobacteriaceae, none of the other genera that constitute the core microbiota of adults are shared with the core microbiota of the pulp or the larvae. Given that the adults studied emerged from puparia in a sterilized cage, these bacteria, likely of environmental origin (pulp of fruit the larvae developed in), must have been transferred from the larvae to the adult via the pupae surviving metamorphosis.

## Discussion

In this study, we pursued the questions of how the microbiota of the larval food is related to the composition of the larval and adult microbiota, and what does this tell us about transience and stability of this species’ gut microbiota and how does metamorphosis affect the adult microbiota by analyzing the microbiota of *A. ludens* developing in six of its hosts. This included two ancestral hosts (*C*. *edulis* and *C*. *greggii*), three exotic hosts (*M. indica*, *P. persica*, and *C. x aurantium*) and one occasional host (*C. pubescens*) that is only used when environmental conditions (such as extreme drought) limit fruiting by the fly’s typical hosts (Thomas, 2004). We deliberately included four highly amendable hosts (*C*. *edulis*, *C*. *greggii*, *M. indica*, and *C. x aurantium*) where the larvae of *A. ludens* develop with little fitness costs, and two hosts in which larvae suffer various fitness costs (*C. pubescens* and *P. persica*; Birke and Aluja, 2018). So, by including these host species, we worked with a gradient spanning all the way from the two ancestral hosts (*C. edulis* and *C*. *greggii*), several exotic but highly suitable hosts, to two natural but sub-optimal hosts (*C. pubescens* and *P. persica*). Notably, the pulp microbiota of the marginal host *C. pubescens* was the most diverse while the pulp of one of the purported ancestral hosts, *C. edulis*, was the least diverse. Furthermore, the microbiota of larvae that developed in *C. pubescens* is also the most diverse, while the microbiota of larvae that developed in the other purported ancestral host, C. *greggii*, was the least diverse.

As can be ascertained in Figure 1, some sampling sites are quite distant from each other. We recognize that ideally, we should have sampled all fruit in a relatively small geographic area to avoid a possible confounding effect between host plant species and geographic site. But this was impossible given biological/ecological considerations. For example, in the case of the two purported ancestral hosts within the Rutaceae, *C. edulis*, and *C*. *greggii*, they very rarely co-occur, and when this is marginally the case, it only happens in highly isolated areas that are not only difficult to reach, but also dangerous because of local drug trafficking. But in the case of *C*. *greggii* and *C. pubescens*/*Mangifera indica*, natural co-occurrence never happens. Based on Liu et al. (2018) and Nikolouli et al. (2020), we are addressing this issue in an additional massive study with *A. ludens* larvae developing in *C.* x *aurantium* a host that has a very wide distribution, which was sampled along a latitudinal and an altitudinal gradient in the state of Veracruz, that is the “longest” state in Mexico, with 803 km separating the northernmost and southernmost borders. In the case of the altitudinal gradient, we sampled all the way from 0 to 100 m above sea level, to 1,800–2,000 m. According to Nobles and Jackson (2020) we could expect an influence of collection site (geography) in larvae but not adults, or vice versa. In contrast, Majumder et al. (2019), working with the tephritid fly *B*. *tryoni*, concluded that “geographic location may play a quite limited role in structuring of larval microbiomes.”

Based on our data, we found that the composition of the gut microbiota of *A. ludens* larvae is plastic rather than stable, depending largely on the microbiota associated with the pulp on which they fed. The proportion of microorganisms in the gut that belong to the resident versus the transient microbiota can vary enormously from one host species to another. For example, some insect species possess a gut microbiota mainly constituted by stable, resident microbiota, as occurs with the American cockroach or *B. tryoni* (Tinker and Ottesen, 2016; Majumder et al., 2019). Other species have a much less stable gut microbiota mainly conformed by transient microbiota, as has been documented in larval or adult butterflies (Hammer et al., 2017; Phalnikar et al., 2019; Ravenscraft et al., 2019a,b), the giant neotropical bullet ant, *Paraponera clavata* or other rainforest ants (Sanders et al., 2017), *Drosophila melanogaster* or other species within *Drosophila* (Wong et al., 2013; Hammer et al., 2017, 2019; Moreau and Rubin, 2017; Ma and Leulier, 2018; Ross et al., 2018; Phalnikar et al., 2019), the cabbage stem flea beetle *Psylliodes chrysocephala* (Shukla and Beran, 2020) or other beetles (Kelley and Dobler, 2011), hard ticks (Guizzo et al., 2020), dragonflies (Deb et al., 2019), and mosquitoes (Coon et al., 2016), but this phenomenon had not been documented previously in the highly polyphagous and pestiferous *A*. *ludens* or any other *Anastrepha* species. In relation to other fruit flies, a study conducted in Australia with *Bactrocera tryoni* (Majumder et al., 2019), indicates that the larval gut microbiota is weakly related to the host fruit microbiota, and rather more strongly associated with vertical transfer of bacteria by females during egg laying. These contrasting results are important, since they suggest that the stability of the gut associated microbiota is not a conserved trait among different fruit fly species. Thus, the degree of plasticity of the gut microbiota among fruit fly species is a topic that deserves further attention. Interestingly, in the case of *B. tryoni* the existence of a high similarity between mycobiome in larvae and their respective host fruit source was documented (Majumder et al., 2020a), suggesting that these fungal communities are closely interconnected, which is in direct contrast with what occurs with bacteria.

The notable differences among the pulp-associated microbiota of the six different *Anastrepha* hosts discovered here, suggest that plants have developed associations with microorganisms along their evolution. So far, few studies have addressed the specificity of fruit microbiota (Dees et al., 2015; Wassermann et al., 2017; Kõiv et al., 2019), but the implication of this specificity could also have important repercussions for plant-insect interactions. In general, there seems to be a consensus indicating that the transient microbiota plays no significant role on the insect’s physiology (Hammer et al., 2017), though notable exceptions exist (Kikuchi et al., 2007; Coon et al., 2016; Heys et al., 2018; Ross et al., 2018). Sometimes the acquisition of microbes from the environment can provide symbionts with functions that are uniquely relevant, including the ability to digest the food which the microbes originally came from Ravenscraft et al. (2019a) and Shukla and Beran (2020).

Here, we provide consistent evidence documenting that pulp-associated microbiota shapes the gut microbiota of *A. ludens* larvae which seems to be mainly transient. But despite this, there are bacterial genera that can have positive or negative effects on larval development, as occurs with mosquitoes (Coon et al., 2016). Given that these transient microorganisms are continuously entering the gut of larvae through their voracious feeding, their positive/negative effects should not be underestimated. Based on our results, perhaps the larvae of *A. ludens* do not develop well in *C. pubescens* and *P. persica* not only because of the intrinsic biochemical composition of their pulp (containing deleterious compounds for the larvae), but also because of the associated microbiota found in the pulp of these fruit. We highlight the fact that the pulp-associated microbiota of *C. pubescens* is clearly different from the microbiota of the other host species (Figures 2, 3, 6). For example, when the pulp microbiota of this fruit was compared with the microbiota of the other five host species, we found that *C. pubescens* is enriched with *Providencia* and *Morganella*, two bacterial genera widely recognized as pathogenic for insects. *Providencia* has been reported as pathogenic for *D*. *melanogaster* and *C. capitata* (Galac and Lazzaro, 2011; Msaad-Guerfali et al., 2018), although this genus is often found in the gut of *A. ludens* adult flies (Kuzina et al., 2001; Nikolouli et al., 2020). On the other hand, *Morganella* is a recognized pathogen for the larvae of this fruit fly species (Salas et al., 2017). So, the role of both genera in the larval development of *A. ludens* in *C. pubescens* must be addressed in the future. In the same comparisons, the microbiota of *C. pubescens* shows under-representation of symbiotic bacteria with positive effects for the development of insects, as is the case of *Acetobacter*. *Acetobacter* is a generalist bacterial genus which usually thrives in carbohydrate-rich environments. Not surprisingly, it is often found in insects developing/feeding on a carbohydrate-rich diet (Crotti et al., 2010). Species within this genus are frequently symbionts of species within *Bactrocera*, such as *B*. *oleae* (Kounatidis et al., 2009) or *B*. *tryoni* (Woruba et al., 2019).

A caveat related to the above is that we did not study the bacteria present in the pulp of uninfested fruit, with no eggs or larvae in them (absolute control). We therefore recognize that our results here need to be confirmed by additional studies contemplating the following two sources of bacteria in the microbiota of *A*. *ludens* guts: (1) microbes naturally present in unaltered fruit; (2) microbes inoculated into the pulp by the mother, the egg surface, or possibly the egg interior. Furthermore, we recognize that the growing larvae could have altered the composition of resident microbes in the pulp via chemical changes, such as introduction of fecal material containing nitrogenous waste. Likely, all three forces may be at play, and this needs to be further investigated.

Since Acetobacteraceae is an over-represented family in the *A. ludens* gut microbiota when it feeds in the pulp of natural hosts where it develops well, this taxon may promote metabolic homeostasis of *A. ludens* by acting as probiotics. Similarly, the families and genera that are over-represented in *C. edulis* with respect to the other hosts also deserve attention, since they could provide new clues about how transient bacteria benefit their hosts. Although the families Weeksellaceae, Acholeplasmataceae, Burkholderiaceae, and the genera *Empedobacter*, *Lampropedia* and *Dysgonomonas* have been previously reported as part of the gut microbiota of *A. ludens* (Salas et al., 2017; Ventura et al., 2018) and other fruit fly species (Martinson et al., 2017; Hadapad et al., 2019; Robert et al., 2019), the description of their association with this ancestral host species is new. In addition, we found that the genus *Leuconostoc* was more abundant in the larvae reared in *P. persica* than in *C. edulis* or *C. pubescens*. Interestingly, in *B*. *tryoni, Leuconostoc* was found to induce a delay in larval development (Shuttleworth et al., 2019) and it could contribute to explain the fitness cost observed in the *A. ludens* larvae reared in this fruit (Birke and Aluja, 2018). Besides, to our knowledge, the family Arcobacteraceae has not been previously described as part of the gut microbiota of *A. ludens*. The few studies on this microorganism have focused on its potential as emergent pathogens in other animal models (Shah et al., 2011; Waite et al., 2017; Chieffi et al., 2020). Since bacteria of the Arcobacteraceae family were present in the *C. edulis* pulp in much higher proportion than in *A. ludens* larvae stemming from this fruit, identifying the species of Arcobacteraceae and their functions could be important for the development of new probiotic formulas for mass rearing of this species. Furthermore, all this could not only imply that *A. ludens* is able to tolerate a wide range of microorganisms in its gut, but also suggests that *A. ludens* larvae are self-sufficient digestors since its functionality does not depend on the co-occurrence of a constant, specific microbiota, as has been previously proposed for insects without a stable microbiota (Hammer et al., 2019). In addition, it is likely that pulp-associated microbiota also promotes or limits the polyphagy of *A. ludens* by adding positive or negative impacts on larval development/fitness. If this is true, a similar dynamic may occur in other plant-insect interactions.

We found that the gut microbiota composition of adult flies (males and females) is remarkably different from that of larvae. This is an important finding, because although the larvae feed on different plants and thus interact with a very heterogeneous microbiota associated to the pulp of the particular host plant they are developing in, adult flies are much less dependent on the plant for their metabolic maintenance. We found a dominance of bacteria within the Enterobacteriaceae in the gut of adults. The dominance of members of this family in the gut microbiota of adults of other species of fruit flies has been widely reported, as is the case of *Bactrocera*, *C. capitata*, *A. obliqua*, or *Zeugodacus tau* (Andongma et al., 2015; Yong et al., 2017; Gallo-Franco and Toro-Perea, 2020; Naaz et al., 2020; Nikolouli et al., 2020, Noman et al., 2021). In contrast, the lack of differences between males and females should come as no surprise, as similar results have been reported for other fruit flies as is the case with *B*. *tryoni* (Woruba et al., 2019; Majumder et al., 2020b), *Zeugodacus cucurbitae* (Hadapad et al., 2019), or *C. capitata* (Nikolouli et al., 2020). According to our data, the relationship between the richness of the gut microbiota of larvae and adults is not constant, because it depends on the particular fruit or substrate where the larvae developed.

Based on our results, metamorphosis plays a key role in shaping the gut microbiota of newly emerged adults. Our findings coincide with other studies that demonstrated sharp differences in the microbiota of immatures and adults of other insects such as butterflies, *Drosophila melanogaster*, or other true fruit flies (i.e., Tephritidae), all documenting a decrease in the diversity of the microbiota in pupae and adults when compared to larvae (Kingsley, 1972; Wong et al., 2011; Hammer et al., 2014; Yong et al., 2017; Malacrinò et al., 2018; Andongma et al., 2019; Majumder et al., 2020b; Nobles and Jackson, 2020). Like us, these authors also found that the microbiota of larvae (in holometabolous insects) or nymphs (in hemimetabolous insects) is influenced by the microbes present in their feeding substrate or surrounding environment and attributed the loss of diversity in pupae and adults to metamorphosis. Kingsley (1972), working with the monarch butterfly (*Danaus plexippus*), was one of the first in suggesting that the loss of diversity in pupae and adults could be due to the major anatomical reconstruction of the alimentary canal during the metamorphosis process and that the larvae could promote the elimination of their bacterial complement to facilitate the introduction and establishment of new bacteria more suitable/amendable for the adults (and their diet). In holometabolous insects that exhibit a complete metamorphosis, the larval gut epithelium is completely replaced during pupation, and during this process, the insect must control the gut microbiota to avoid septicemia and at the same time avoid the loss of beneficial mutualists (Johnston and Rolff, 2015). In lepidopterans it has been reported that within the newly formed gut epithelium immune effectors or lysozyme are synthetized before delamination of the larval epithelium, which are discharged into the lumen when the epithelium is complete as a prophylactic response to protect the insect from microbial infections (Russell and Dunn, 1991, 1996; Johnston et al., 2019). Recently, Huang et al. (2019) studying the microbial communities in different developmental stages of *B. dorsalis* found that microorganisms in larvae and adults are primarily Gram-negative and the major components in pupae are Gram-positive bacteria. They suggested that maintenance of the microbiota in the different developmental stages is associated with expression of the genes encoding peptidoglycan recognition proteins (PGRP-LB and PGRP-SB1). In the case of *A. ludens*, all the latter still needs to be studied. However, based on detailed recent studies on the gut of this insect (Guillén et al., 2019), we suggest that flies could recover important bacteria from other structures linked to the gut that harbor microbes such as the dorsal esophageal bulb and crop (Lloyd et al., 1986; Stoffolano, 2019) that despite suffering structural changes “survive” metamorphosis (Chapman, 1998). We note, that *A. ludens* adults have a very different diet when compared to larvae and that when they emerge from the puparia after metamorphosis, they need to quickly find nutrients for survival. Perhaps the larval microbiota that persists in the adult gut could help newly emerged flies extract the first critical nutrients from the various feeding substrates commonly used [honeydew, bird feces, rotting pulp, or liquids oozing from decomposing fruit (Aluja et al., 2000)], while they acquire the “adult microbiota” (Drew et al., 1983; Drew and Yuval, 2000; Morimoto et al., 2019). For adults, protein is necessary to reach sexual maturity and develop healthy ovaries in females and testes in males (Aluja et al., 2001). All this needs to be studied in the future.

In a previous study, it was reported that the gut microbiota of *A. ludens* adults is more diverse than the microbiota of gut larvae (Ventura et al., 2018) which contrasts with our findings here. This inconsistency could be due to the method used to sample adults. In our case, we used newly emerged flies because we wanted to know if flies emerged with a core microbiota. In the case of Ventura et al. (2018), they used flies captured in Multilure® traps baited with CeraTrap®, a liquid protein-based attractant where flies were trapped. Fly aspects that could influence microbiota diversity as the age or the life history (e.g., types of food consumed) were unknown; besides, since flies were immersed in the liquid bait, they could have acquired an unnatural load of microorganisms from it. In fact, this was the reason why we left out adults collected in the field in the plants we sampled as they are highly mobile and therefore could have picked up bacteria from feeding [rotten fruit (likely containing yeasts, fungi, and bacteria), bird feces, honey dew, protein baits used in traps] and mating/resting sites (leaves) that had little to do with metabolizing the pulp ingested by larvae inside fruit or aiding in the process. In future studies such as the one by Ventura et al. (2018), more attention must be paid to possible sources of off-target microorganisms to avoid being misled.

Finally, one of our goals here was to determine if there was a “core microbiota” in the larvae of *A*. *ludens* infesting various hosts. In this sense, the comparison between the two ancestral hosts, *C*. *edulis* and *C*. *greggii* (both Rutaceae) was highly pertinent. The composition of the microbiota of larvae developing in these two hosts was remarkably different, with little overlap, except for bacteria within the Acetobacteraceae and Enterobacteriaceae. As noted, before, with few exceptions, the geographical distribution of these two ancestral hosts does not overlap. So, as we found that the gut of larvae principally harbors bacteria accrued from the pulp they fed on, possibly over evolutionary time, these two host plants developed significantly different associations with bacteria, likely related to fitness advantages to the plant, not the fruit fly larvae developing in them. But broadly speaking, and as clearly depicted in Figure 8, we were unable to find an important core microbiota in neither larvae nor adults stemming from six different hosts encountered in nature. In fact, the estimated core microbiota of larvae is equivalent to that of the microbiota associated to the pulp. However, these results do not imply that the bacterial groups found as constituents of the core microbiota are not important. In fact, it is likely that these taxa represent preponderant players for the larval development of *A. ludens*. It is noteworthy that most of the very small core microbiota estimated for larval gut samples did not share elements with the estimated core microbiota of adult flies, with exception of the bacteria that belong to the Enterobacteriaceae. However, even in this case, the ubiquity of the members of Enterobacteriaceae could explain these results. Additional studies are needed to confirm if the identified bacterial species in adults stem from previous development stages (i.e., larvae or pupae) or are derived from the environment via feeding activities by newly emerged adults.

## Conclusion

We found that the gut microbiota of *A. ludens* is unstable and that its composition and richness depend on the microbiota associated with the particular plant the larvae of this highly polyphagous species fed on. The pulp of each host plant harbors a specific microbiota, which may affect the compatibility of *A. ludens* with a particular fruit, as is the case of the occasional host *C. pubescens*, in which we found a significant presence of *Providencia* and *Morganella*, two genera of bacteria that are known to be pathogenic for insects. In this sense, we failed in our prediction that *A*. *ludens* larvae would harbor a core microbiota providing flies with a broad community of bacteria useful in metabolizing undigestible or toxic chemicals in the fruit pulp. However, the apparent lack of a stable microbiota in the larval stage in *A. ludens* paves the way to researching which biochemical mechanisms and metabolic pathways *A*. *ludens* larvae use to degrade toxic chemicals in the pulp or supplement deficient larval diets in the field. Finally, and more broadly (i.e., beyond tephritid flies), our findings add new perspectives on the role that metamorphosis plays in shaping the gut microbiota present in insect adults.

## Data Availability Statement

The datasets presented in this study can be found in online repositories. The names of the repository/repositories and accession number(s) can be found in the article/Supplementary Material.

## Author Contributions

MA, LG, and AM: experimental design. AA-M: sample collection. AA-M and AA-S: experimental procedures. JAZ-B and MA: writing. JAZ-B, VP-B, DD, and MV-R-L: data analysis. JAZ-B: formal analysis. JAZ-B, MA, LG, AA-M, AM, VP-B, DD, MV-R-L, and EI-L: editing. MA and AM: supervising and founding resources. All authors contributed to the article and approved the submitted version.

## Conflict of Interest

The authors declare that the research was conducted in the absence of any commercial or financial relationships that could be construed as a potential conflict of interest.

## Publisher’s Note

All claims expressed in this article are solely those of the authors and do not necessarily represent those of their affiliated organizations, or those of the publisher, the editors and the reviewers. Any product that may be evaluated in this article, or claim that may be made by its manufacturer, is not guaranteed or endorsed by the publisher.

## Footnotes

1
https://github.com/hrbrmstr/ggalt

2
https://CRAN.R-project.org/package=microbial

3
http://www.R-project.org/

## Supplementary Material

Supplementary Figure 1 Rarefaction curves for each host fruit considering microbiota in fruit pulp, and larval and adult (female and male) guts.

Supplementary Figure 2 Alpha diversity in the microbiota identified in each host fruit (considering the fruit pulp, and *Anastrepha ludens* larvae and adults) estimated with the Shannon index. Upper and lower whiskers, 3rd, and 1st quartiles (top and bottom of the box), and median (horizontal line within the box) are displayed. Asterisks indicate significant differences between the pairs connected by the horizontal line. For paired *t*-test, *, **, *** represent *p* < 0.05, 0.01, and 0.001, respectively.

Supplementary Figure 3 Linear discriminant analysis (LDA) effect size (LEfSe) results showing the significant results (horizontal bars) and the differential taxa for larvae gut microbiota among host plants. Each bar represents the enriched bacterial taxa in the larvae microbiota from the corresponding host plant when it is compared with other host plants. F, family; g, genera. Only taxa with at least family annotation were plotted.

Supplementary Table 1 Details on *A. ludens’s* hosts used in study as well as on collection sites in different states of Mexico.

Supplementary Table 2 Number of raw sequences per sample, after filtering, merging, and removing chimeras.

Supplementary Table 3 Abundance of ASVs and their annotation detected across the libraries. We only report taxa with >1% relative abundance across the dataset.
