# Supplementary figures and images for: Metagenomic Survey of the Highly Polyphagous Anastrepha ludens Developing in Ancestral and Exotic Hosts Reveals the Lack of a Stable Microbiota in Larvae and the Strong Influence of Metamorphosis on Adult Gut Microbiota
Source: Front Microbiol. 2021 Aug 2;12:685937. doi: 10.3389/fmicb.2021.685937 (PMC8367737; doi:10.3389/fmicb.2021.685937)

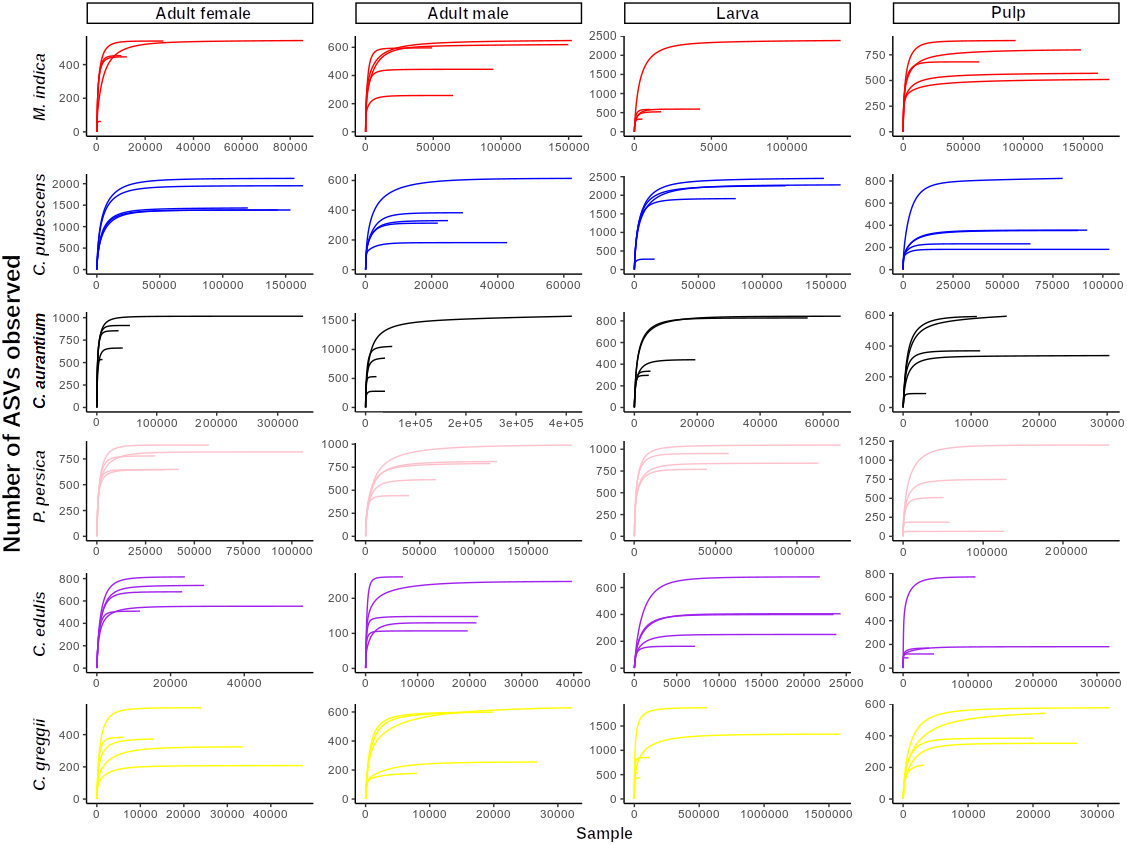

Supplement: Supplementary Figure 1 — Rarefaction curves for each host fruit considering microbiota in fruit pulp, and larval and adult (female and male) guts. [file Image_1.tiff]

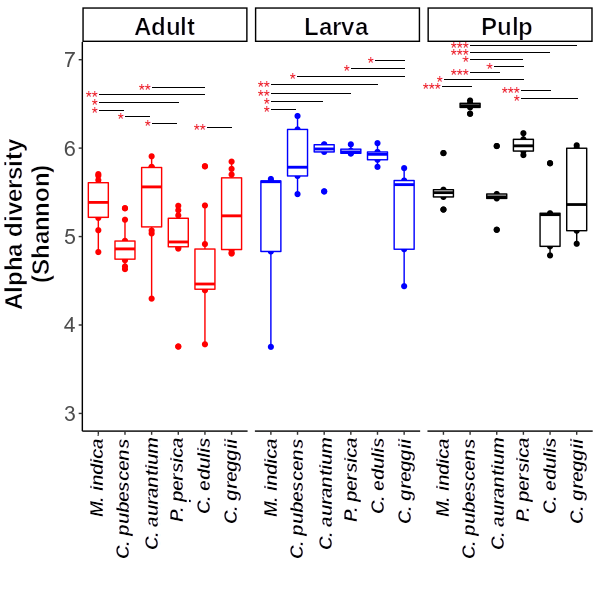

Supplement: Supplementary Figure 2 — Alpha diversity in the microbiota identified in each host fruit (considering the fruit pulp, and Anastrepha ludens larvae and adults) estimated with the Shannon index. Upper and lower whiskers, 3rd, and 1st quartiles (top and bottom of the box), and median (horizontal line within the box) are displayed. Asterisks indicate significant differences between the pairs connected by the horizontal line. For paired t-test, *, **, *** represent p < 0.05, 0.01, and 0.001, respectively. [file Image_2.tiff]

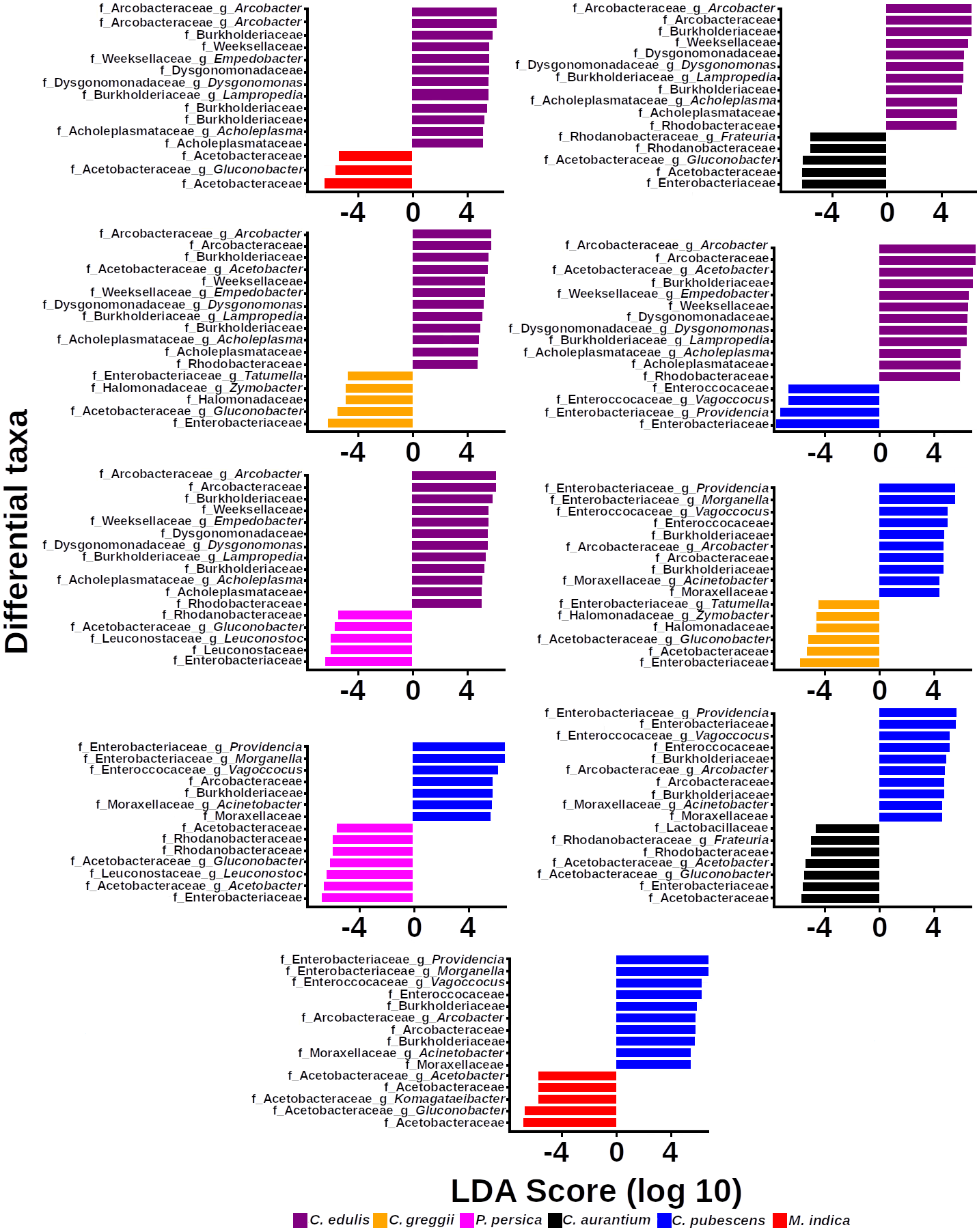

Supplement: Supplementary Figure 3 — Linear discriminant analysis (LDA) effect size (LEfSe) results showing the significant results (horizontal bars) and the differential taxa for larvae gut microbiota among host plants. Each bar represents the enriched bacterial taxa in the larvae microbiota from the corresponding host plant when it is compared with other host plants. F, family; g, genera. Only taxa with at least family annotation were plotted. [file Image_3.tiff]
